# A Rare Finding of Gastric Pseudomelanosis in Chronic Kidney Disease: A Case Report

**DOI:** 10.7759/cureus.38933

**Published:** 2023-05-12

**Authors:** Mohamed Ibrahim, Michael Herman

**Affiliations:** 1 Osteopathic Medicine, Lake Erie College of Osteopathic Medicine, Bradenton, USA; 2 Gastroenterology, Borland Groover, Jacksonville, USA

**Keywords:** drug-induced pigmentation, iron supplement, chronic kidney disease (ckd), duodenal melanosis, pseudomelanosis

## Abstract

Pseudomelanosis is a black-brown discoloration of the loose connective tissue layer of the intestinal mucosa, also known as the lamina propria. Although it is a benign condition and poses no real threat to the patient, it has been known to be associated with certain medication use in the colon, like anthraquinone laxatives, as well as various chronic illnesses in the duodenum and stomach, like iron deficiency anemia, end-stage kidney disease, hypertension, and diabetes mellitus. Only a handful of cases of gastric pseudomelanosis have been reported in the literature, often presenting to the physician as an elderly female with dark, tarry stools from excessive iron use. In this unusual case, a 75-year-old male came to the emergency room due to a concern about blackish stools in the toilet. After reviewing his past medical history, it was found that he takes iron tablets for anemia secondary to end-stage renal disease. While enteric iron was most likely the cause of the melena, an esophagogastroduodenoscopy (EGD) study was performed to rule out any proximal causes of gastrointestinal bleeding. Following the upper endoscopy, gastric pseudomelanosis was established.

## Introduction

Pseudomelanosis is defined as a benign, dark, speckled pigmentation within the subepithelial macrophages of the lamina propria layer in the enteric wall [1]. While it is most commonly found in the colon and associated with anthraquinone laxative use, cases have been reported in the small intestine as well as the stomach, with the incidence significantly decreasing more proximally [2]. The incidence in the large intestine has been reported between 0.82% and 1.13%, with a staggering 95% of those patients disclosing they take anthraquinone laxatives [2]. On the contrary, to the best of our knowledge, only eight other reported cases of gastric pseudomelanosis were previously found in the literature [3, 4]. There does seem to be a correlation between this condition and other chronic diseases that we see in our patients as well, such as diabetes mellitus, hypertension, iron deficiency anemia, chronic kidney disease, gastrointestinal bleeding, and hemochromatosis [3]. We present the case of a 75-year-old male who arrived at the emergency department from a rehabilitation center after noticing dark, tarry stools in the toilet. After further investigation and an esophagogastroduodenoscopy (EGD) study, pseudomelanosis of the gastric greater curvature was confirmed.

## Case presentation

A 75-year-old male came to the emergency department via emergency medical services (EMS) from a rehabilitation center due to a large, dark, tarry stool prior to arrival. He became significantly hypotensive after the bowel movements, which all occurred after a dialysis session. He denied any chest pain, shortness of breath, abdominal pain, nausea, vomiting, fever, or chills. He had never had an esophagogastroduodenoscopy (EGD) and reported normal colonoscopy screenings in the past. He also denied any use of non-steroidal anti-inflammatory drugs (NSAIDs). He has a past medical history of end-stage renal disease (ESRD) on hemodialysis, hypertension, hyperlipidemia, cerebrovascular accident (CVA), iron deficiency anemia, gastroesophageal reflux disease (GERD), and type 2 diabetes mellitus. His laboratory values are listed below in Table 1.

**Table 1:** Laboratory values mEq/L: milliequivalents per liter; mg/dL: milligrams per deciliter; mL/min: milliliters per minute; g/dL: grams per deciliter; K/mcL: thousands per microliter; U/L: units per liter

Laboratory test	Value	Reference range
Sodium	137 mEq/L	135-146 mEq/L
Potassium	4.5 mEq/L	3.5-5.3 mEq/L
Chloride	100 mEq/L	98-110 mEq/L
Carbon dioxide (CO2)	27 mEq/L	23-29 mEq/L
Blood urea nitrogen (BUN)	64 mg/dL (High)	7-25 mg/dL
Creatinine	6.0 mg/dL (High)	0.6-1.2 mg/dL
Estimated glomerular filtration rate (eGFR)	<15 mL/min (Low)	>60 mL/min
Glucose	106 mg/dL	70-110 mg/dL
Calcium	7.5 mg/dL (Low)	8.4-10.2 mg/dL
Albumin	2.9 g/dL (Low)	3.5-5.5 g/dL
Phosphorous	4.5 mg/dL	3.0-4.5 mg/dL
Magnesium	2.3 mg/dL (High)	1.5-2.0 mg/dL
White blood cells	15.70 K/mcL (High)	4.5-11 K/mcL
Hemoglobin	6.9 g/dL (Low)	13.5-17.5 g/dL
Hematocrit	20.2% (Low)	41-53%
Platelets	173 K/mcL	150-400 K/mcL
Alkaline phosphatase	60 U/L	25-100 U/L
Total bilirubin	0.5 mg/dL	0.1-1.0 mg/dL
Total protein	5 g/dL (Low)	6.0-7.8 g/dL
Alanine transaminase (ALT)	6 U/L (Low)	10-40 U/L
Aspartate aminotransferase (AST)	8 U/L (Low)	12-38 U/L

**Figure 1:**
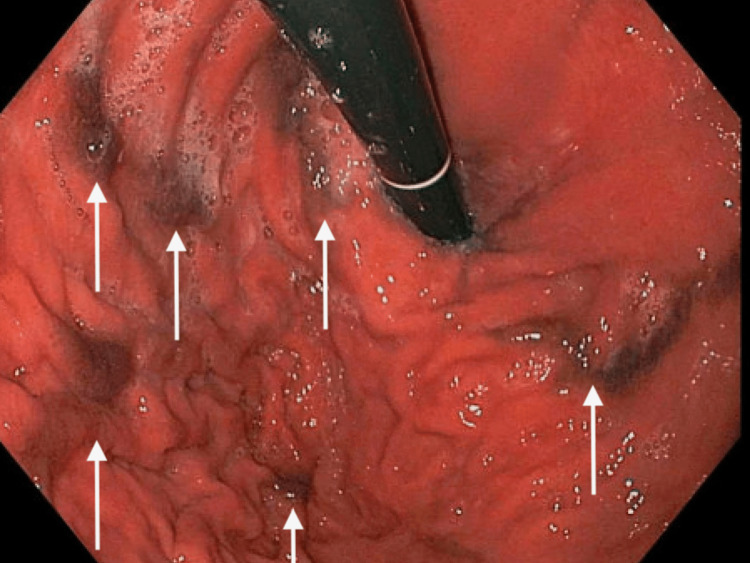
Esophagogastroduodenoscopy demonstrating the pigmented lesions in the greater curvature of the stomach

**Figure 2:**
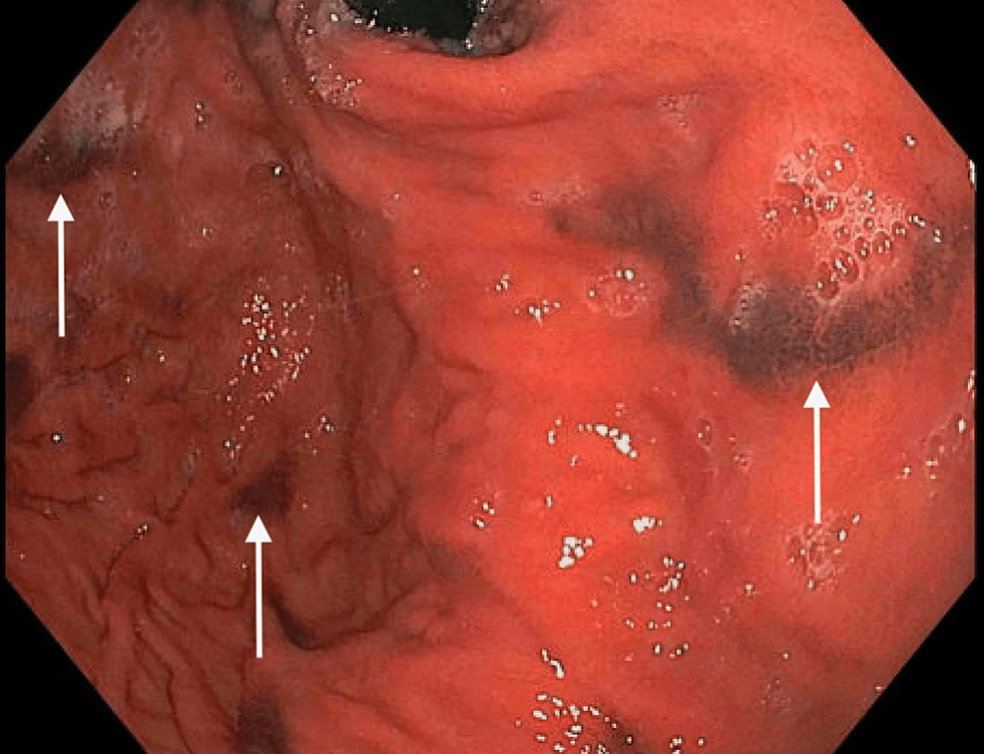
Esophagogastroduodenoscopy demonstrating the pigmented lesions in the greater curvature of the stomach

His abnormal lab values were all attributed to his end-stage renal disease due to the kidney’s inability to filter out urea and creatinine given the low glomerular filtration rate. The kidneys also produce vitamin D, which is needed to properly absorb calcium, hence the low calcium levels, and they also produce erythropoietin, which is needed to stimulate red blood cell production, hence the low hemoglobin and low hematocrit. The basement membrane in the kidneys is also responsible for not allowing proteins such as albumin to pass into the urine, but in ESRD, that membrane is disrupted, and there is a significant loss of proteins in the urine. Although the anemia was likely due to kidney failure, an esophagogastroduodenoscopy study had to be performed to rule out a possible upper gastrointestinal bleed. The endoscopy evaluation in Figure 1 and Figure 2 showed friable, discolored mucosa with scattered areas of pigmented spots within the gastric antrum and distal body of the stomach.

Gastric biopsies of the areas were taken and sent to the pathology lab. Histopathology revealed normal oxyntic mucosa with superficial deposits of iron (confirmed with Prussian blue iron staining), surface erosion, and associated reactive changes. There was no *Helicobacter pylori*, intestinal metaplasia, dysplasia, or malignancy identified. The pathologist commented that superficial deposits of iron are commonly associated with oral iron supplementation, and unmanaged iron intake can lead to significant ulceration and bleeding. The patient became stable in the hospital after the endoscopy and continued hemodialysis with an increase in hemoglobin and hematocrit. He continued to improve clinically and was cleared for discharge with instructions to avoid NSAIDS, increase dietary fiber, and continue his iron supplementation upon discharge.

## Discussion

Gastric and duodenal pseudomelanosis are very rare and benign findings that are most often found incidentally on endoscopy following a complaint of abdominal pain, hematemesis, hematochezia, or melena [5]. The common theme amongst the few case reports that have been documented was the presence of iron in the tips of the lamina propria within the greater curvature of the stomach, or duodenal villi [6]. While iron was always the main culprit, traces of calcium, aluminum, magnesium, potassium, and other elemental ions have also been identified [6]. The main diagnostic method for this condition is a Prussian blue stain that can visualize the hemosiderin in the mucosa by releasing ferric iron (Fe3+) after the cells are treated with hydrochloric acid [7]. The metal then reacts with potassium ferrocyanide to form ferric ferrocyanide, which radiates as a bright blue pigment [7].

It should be noted that melanosis has been well-reported in the colon and rectum [8]. Although melanocytes are not present in the gastrointestinal tract, some pigmented lesions in the large intestine do contain melanin and lipofuscin, hence the name melanosis coli [8]. On the other hand, the discoloration that has been seen in the duodenum and stomach is a heterogeneous mixture of iron, hemosiderin, and small amounts of lipofuscin with no melanin present at all, hence the name pseudomelanosis [8]. While pseudomelanosis has been recorded throughout the entire enteric system, melanosis has strictly been focused on the colon and rectum [8].

While this pigmentation is very rare in the lining of the stomach, there has been a conservatively higher incidence in the small intestine. In a Portuguese facility that had performed over 600 capsule endoscopies and 100 double balloon endoscopies, there were only two cases of small bowel pseudomelanosis, with one patient demonstrating discoloration throughout the entire small bowel and one patient having it localized to the ileum [9]. In Korea, there have been seven randomly reported cases of pseudomelanosis in the duodenum and ileum, but the interesting fact was that all the patients had been consuming iron supplements, and no organic pathology was indicated as the cause [9]. In the eight known cases of gastric pseudomelanosis, all but one patient were female, with an average age of 73.4 years, making our male patient an anomaly [1]. One consistent factor among all eight patients is that they were taking a ferrous sulfate pill for iron deficiency anemia secondary to chronic kidney disease [1]. While 70% of people who take anthraquinone laxatives for constipation or other chronic conditions develop pseudomelanosis in their colon, there is no definitive data or enough literature to identify the root cause of pseudomelanosis elsewhere in the gastrointestinal tract [9]. The limited availability of case reports and case series may suggest that persistent use of ferrous sulfate supplements in those with renal failure might predispose one to develop gastric pseudomelanosis.

Unfortunately, the literature is very limited when trying to hypothesize the risk factors and etiology associated with this benign condition. The diagnosis of pseudomelanosis in the upper gastrointestinal tract could become more prevalent as the elderly population increases with the generation of baby boomers approaching their late 60s and early 70s. For those who are being treated for chronic metabolic diseases like renal failure with iron supplements for anemia, physicians should consider the side effects of these medications and warn patients about the possibility of dark, tarry stools. It should be at the top of the differential list for this population group when they present to the emergency room with these types of symptoms. This case report emphasizes the importance of a comprehensive past medical history and medication list in the hospital setting, which can help avoid unnecessary intensive abdominal workups. We urge clinicians to have a more broad-based approach to their practice of medicine and perform a thorough review of the patient's chart, as it can be very beneficial and helpful in determining a diagnosis.

## Conclusions

Gastric pseudomelanosis is a harmless presentation of chronic ferrous sulfate use that was once speculated to mainly occur in elderly females. The storage form of iron, hemosiderin, deposits in the lamina propria of the gastric mucosa as well as the bulbs of the proximal duodenal villi, displaying areas of blackish-brown pigmentation on an esophagogastroduodenoscopy (EGD) study. A routinely seen characteristic of the finite number of patients with this condition is that the majority had chronic kidney disease as well as many other comorbidities such as diabetes mellitus, hypertension, anemia, hemochromatosis, and bouts of gastrointestinal bleeding. Physicians, particularly nephrologists and gastroenterologists, should be aware of the potential for pseudomelanosis in the stomach secondary to chronic iron supplementation due to renal failure-associated anemia, especially when presented with a patient with unexplained melena, or black, tarry stools.
